# Supplementary material for: Feasibility of Genipin to Evaluate Chitosan Rainfastness for Biopesticide Applications
Source: Int J Mol Sci. 2025 Jan 25;26(3):1031. doi: 10.3390/ijms26031031 (PMC11816675; doi:10.3390/ijms26031031)
Supplement: Supplementary file 1 [file ijms-26-01031-s001.zip › ijms-3402594-supplementary.pdf]

Supplementary Materials

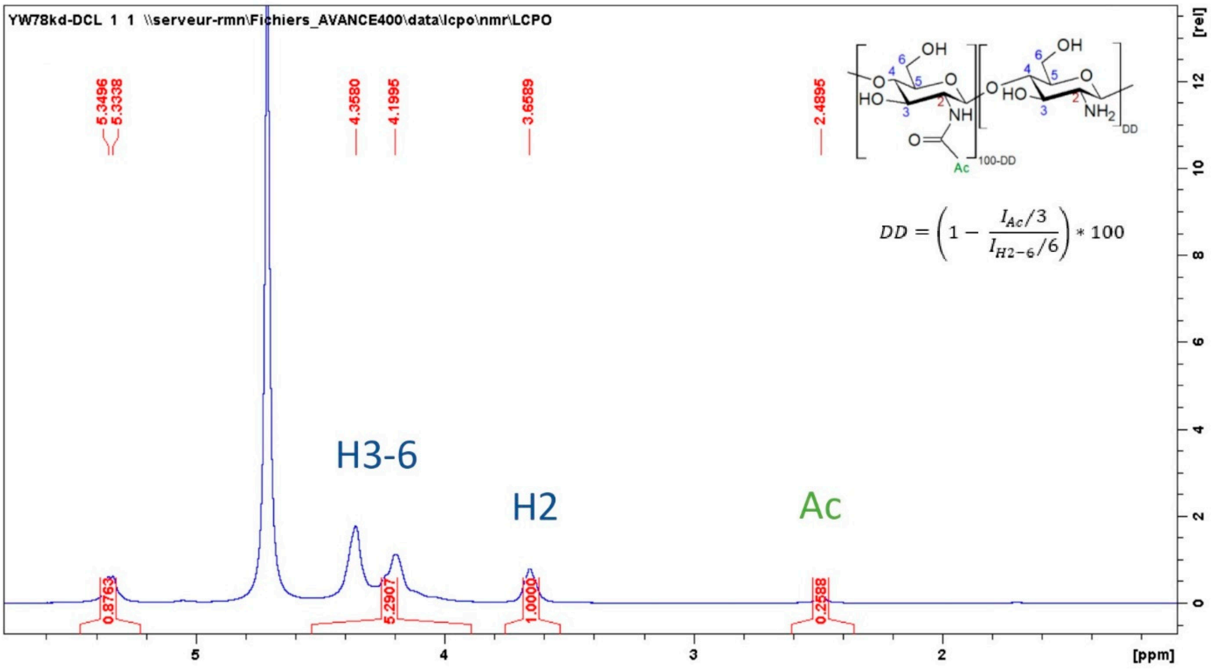

Figure S1. H1 NMR spectra with attributions and DD equation.

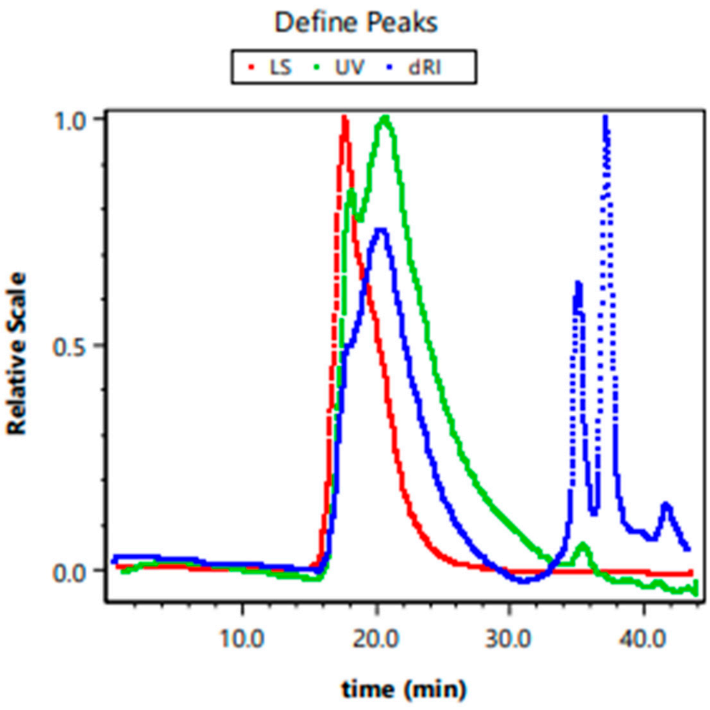

Figure S2. SEC of the chitosan sample with UV, Light Scattering and differential Refractive Index spectrum.

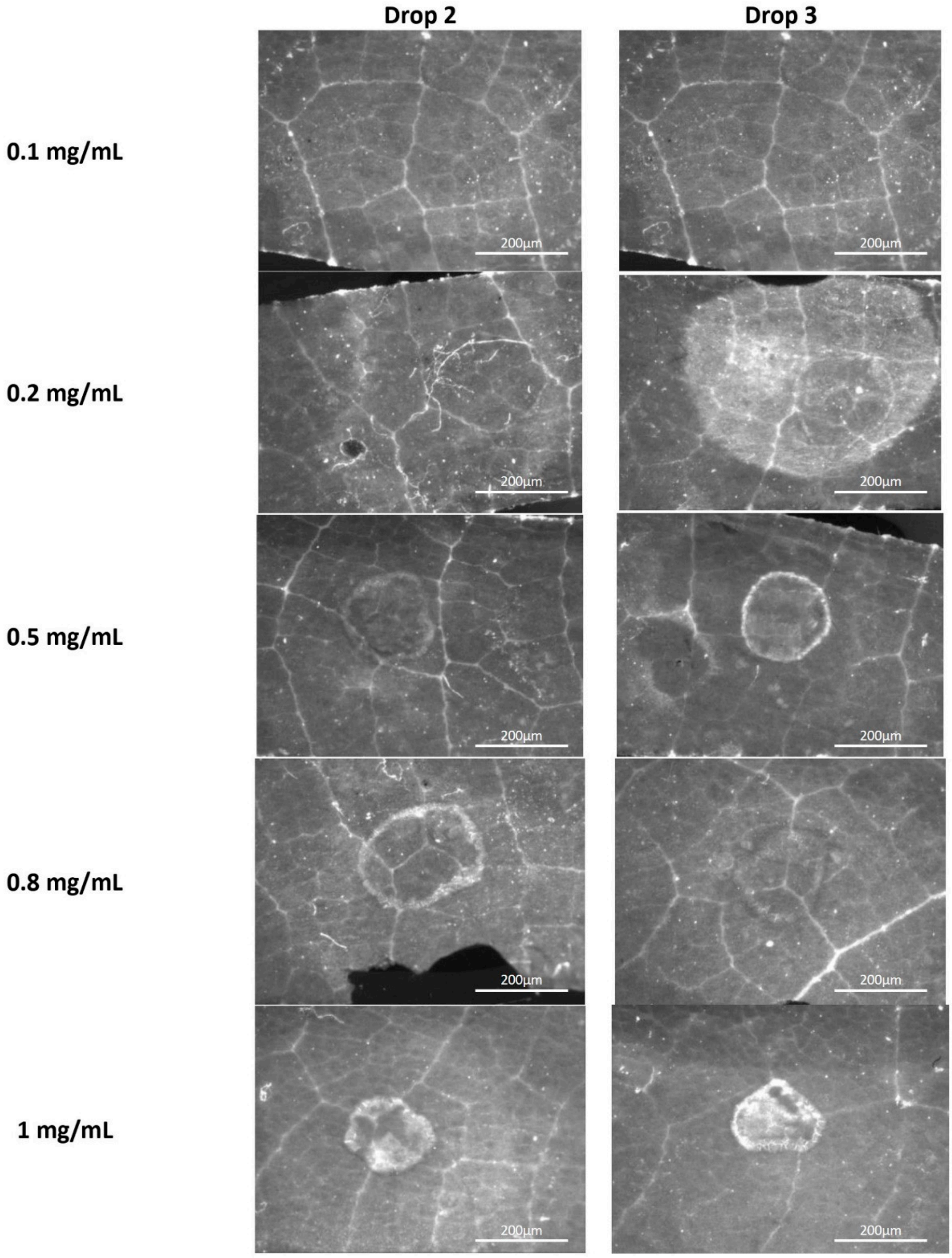

Figure S3. MacroFluo of chitosan between 0.1 and 1 mg/mL.

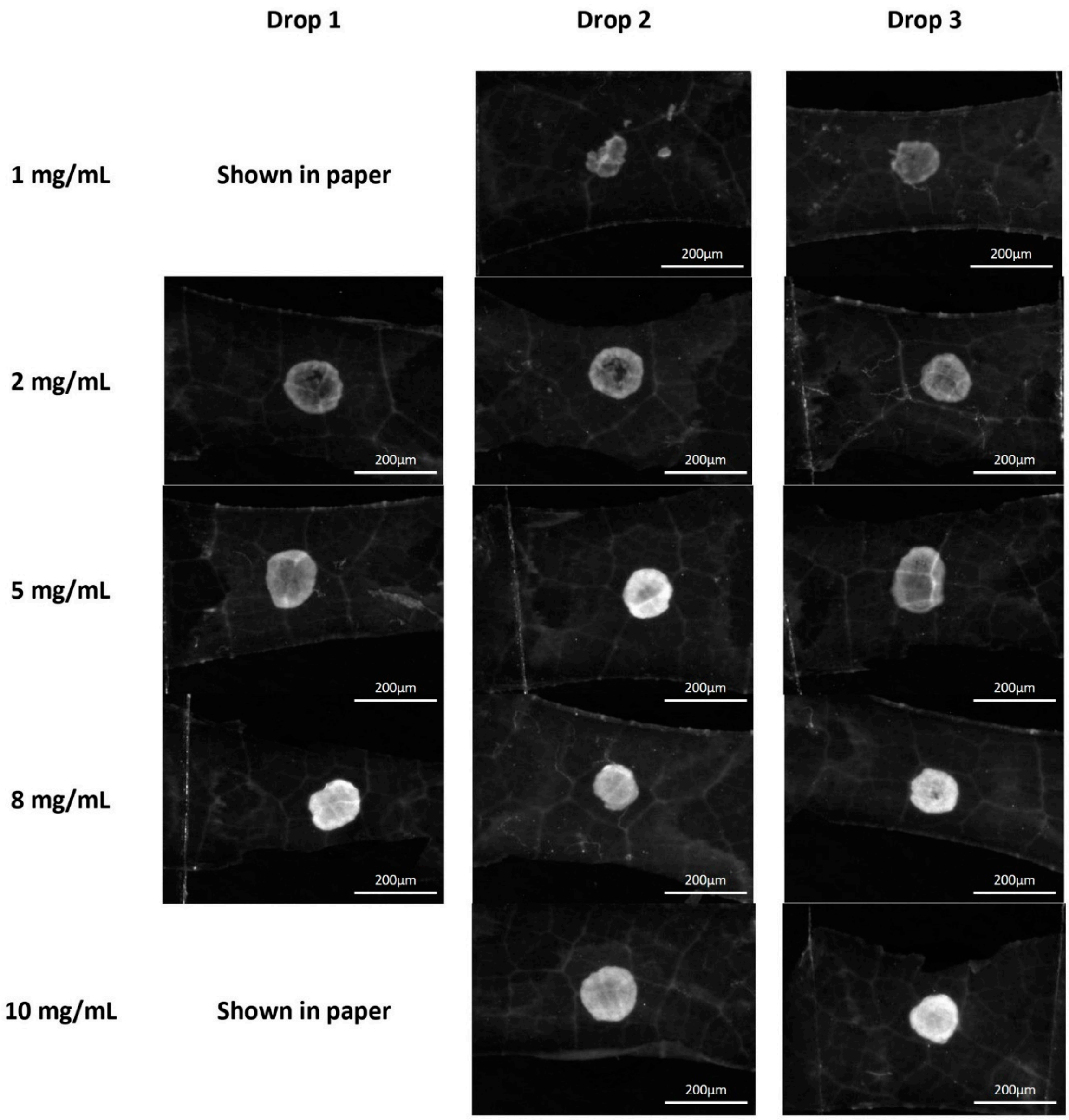

Figure S4. MacroFluo of chitosan between 1 and 10 mg/mL.

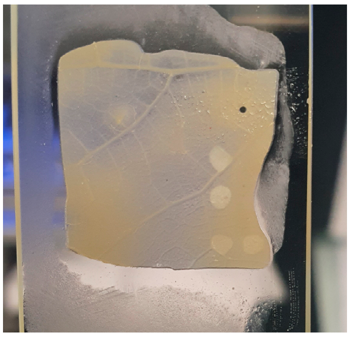

Figure S5. Carnauba wax model after water drops deposition.

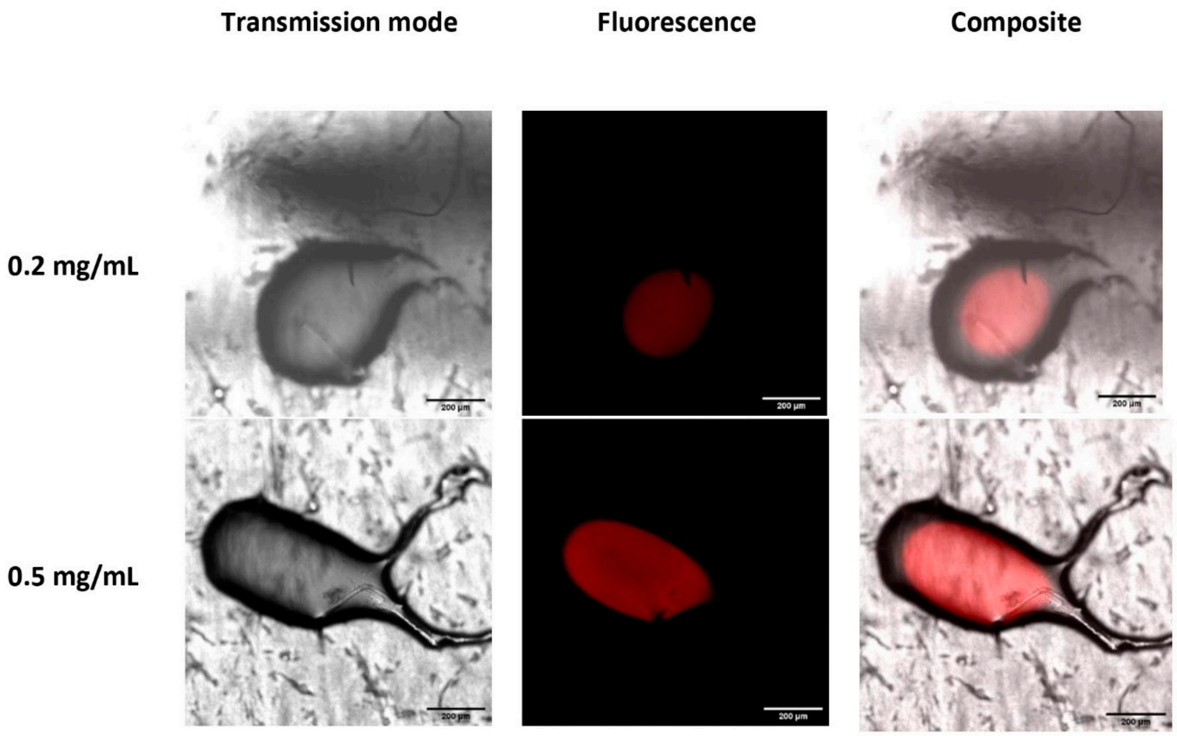

Figure S6. CLSM of chitosan at different concentration in citric acid.

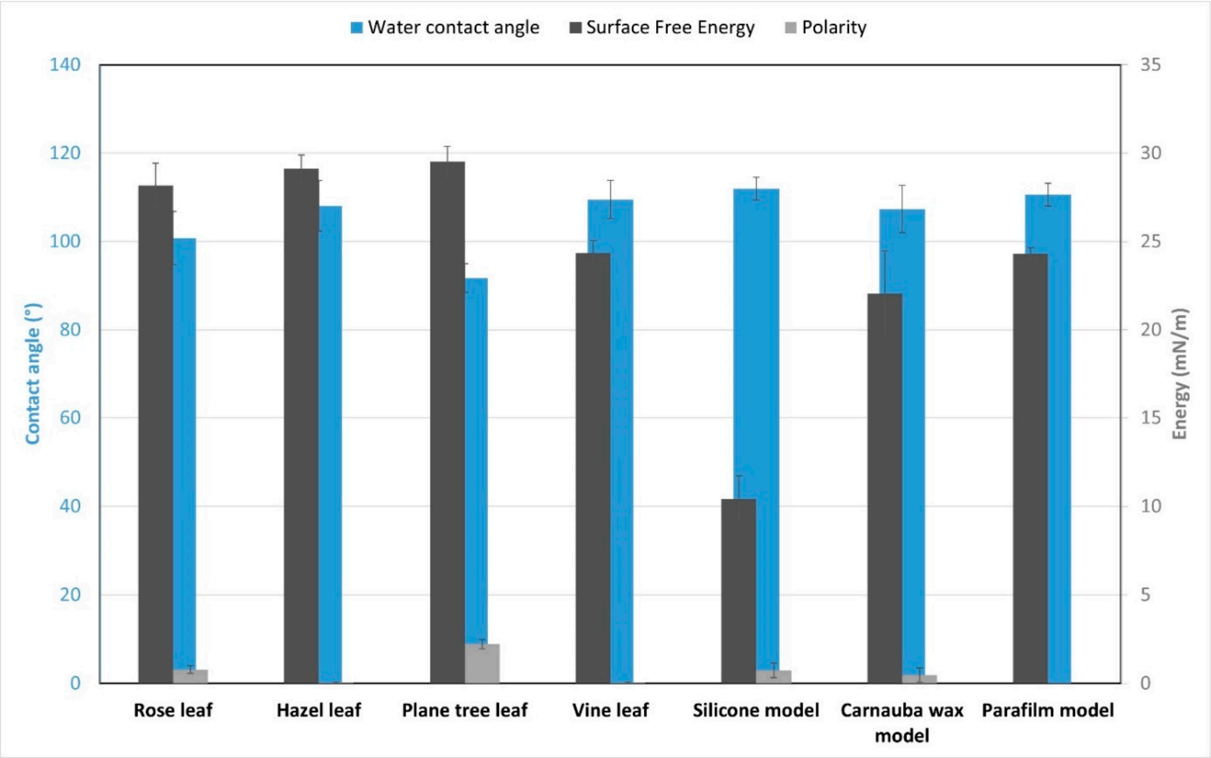

Figure S7. Water contact angle (grey) and Surface Free Energy (black) comparison of leaf models and real leaves from the surrounding area of our lab or from field. Leaves other than were collected, washed and cut into 2x0.5cm strips before being put onto a microscope slide.
